# Bottom‐Up Ice Growth Geometry Attenuates Shear Stress and Improves the Cryopreservation of Hematopoietic Stem/Progenitor Cells Under Low DMSO Concentrations

**DOI:** 10.1002/bit.70116

**Published:** 2025-11-28

**Authors:** Rafaela Ouro Neves, Pedro Sena Rego, Marta H. G. Costa, Isabel Bogalho, Andreia Duarte, Claúdia L. da Silva, Frederico Castelo Ferreira, Vitor Geraldes, Miguel A. Rodrigues

**Affiliations:** ^1^ SmartFreez, Ed. Inovação II Incubadora do Taguspark Porto Salvo Portugal; ^2^ Department of Bioengineering and Institute for Bioengineering and Biosciences (iBB), Instituto Superior Técnico Universidade de Lisboa Lisboa Portugal; ^3^ Associate Laboratory i4HB ‐ Institute for Health and Bioeconomy, Instituto Superior Técnico Universidade de Lisboa Lisboa Portugal; ^4^ Centro de Química Estrutural, CQE, Instituto Superior Técnico Universidade de Lisboa Lisboa Portugal

**Keywords:** bottom‐up ice growth, computational fluid dynamics, cryopreservation, DMSO, freezing geometry, hematopoietic stem/progenitor cells

## Abstract

Studies on cell cryopreservation have been limited by the complexity of the freezing process and challenges on controlling ice formation, managing cooling rates, and optimizing cryoprotectant concentrations. The objective of this study is to evaluate the impact of bottom‐up and conventional radial freezing on the viability of mammalian cells, using mouse hybridoma cells and human umbilical cord blood (hUCB) derived mononuclear cells (MNCs) as models. UCB‐derived MNCs were selected for this study because these contain hematopoietic stem and progenitor cells (HSPCs), which hold significant clinical relevance. The study combines experimental assays, including cell viability assays and flow cytometry characterization, with Computational Fluid Dynamic (CFD) simulations. A bottom‐up freezing geometry sustained high cell viability, even at dimethyl sulfoxide (DMSO) concentrations below 5% (v/v), whereas conventional radial freezing led to lower cell viability when DMSO is used below such concentrations threshold. This observation is particularly relevant for cell‐based therapies. CFD simulations for conventional radial freezing elucidated that for such method the ice formed at the top of the vial is of high porosity for media with 10% (v/v) DMSO, but of low porosity for lower DMSO concentrations. The simulations show that the latter conditions can result in an increase in shear stress on cells, by up to an order of magnitude. Overall, this study provides a rational for 10% (v/v) DMSO being the optimal reported concentration for conventional freezing methods, as a result of poor control of ice growth direction and higher mechanical stresses at lower DMSO concentrations. Experimental results show that bottom‐up freezing method, using only 2.5% (v/v) DMSO, allow to reach cell viabilities as high as the ones obtained with conventional radial freezing protocols at 10% (v/v) DMSO. In addition, bottom‐up freezing method with 2.5% DMSO preserves the clonogenic potential of HSPCs within hUCB‐derived MNCs comparably to conventional radial freezing protocol with 10% DMSO. Importantly, the results support the recommendation to use cell cryopreservation strategies, such as bottom‐up freezing, that enable the use of lower DMSO concentrations by controlling the direction of heat transfer.

## 1 Introduction

Transplantation of hematopoietic stem/progenitor cells (HSPC) is currently used to treat haemato‐oncological diseases. The use of off‐the‐shelf and easily accessible HSPC‐based products in clinical settings requires the development of reliable cryopreservation techniques suitable for cell storage and transportation (Kemp 2006; Martin et al. 2006; Meneghel et al. 2020). The general scaling‐up of cell therapies has highlighted important shortcomings of current cryopreservation technologies. Issues related with low cell survival, low therapeutic potency, cell epigenetic modifications, and limited process scalability can pose significant threats to the consistency and widespread adoption of cell therapies (Burand et al. 2017; Eapen et al. 2020; Maurer et al. 2021; Pollock et al. 2017; Singh et al. 2017).

The use of cryoprotectant agents is essential to mitigate some of the challenges encountered during cell cryopreservation. Dimethyl sulfoxide (DMSO) has been the critical agent to prevent ice nucleation inside the cells and moderate osmotic pressure imbalances during freezing and thawing. However, it can also potentiate several adversities, including toxicity to the patients once DMSO‐cryopreserved cells are infused (Fry et al. 2015; Hunt 2019; MAZUR 1963; Shu et al. 2014; Zenhäusern et al. 2000). Several studies show the need to decrease DMSO concentration while freezing, thus reducing toxicity to the patient upon cell therapy administration, without compromising high survival rate and functionality of cells upon thawing (Gilfanova et al. 2021; Haastrup et al. 2021; Mitrus et al. 2018; J. P. Rodrigues et al. 2008). Haastrup et al. (2021) successfully froze T cells using just 1% to 2% DMSO in combination with pentaisomaltose, an approach that showed enhanced cell proliferation and migration after thawing. However, they discovered that the cryoprotective efficiency of pentaisomaltose and DMSO varies, depending on the cell type. Gilfanova et al. (2021) also demonstrated that cryopreserving hematopoietic stem cells with a 5% DMSO solution was equally effective as using a 10% DMSO solution; however, lower DMSO concentrations were not evaluated.

The search for alternatives to DMSO has led to the examination of a wide range of compounds, such as sugars, proteins, polymers, amino acids, and various small molecules and osmolytes. Moreover, several techniques have been explored to enhance the efficient uptake of these cryoprotectants by cells (Yao and Matosevic 2021). Recently, Kaushal et al. (2022) assessed four DMSO‐free cryopreservation solutions and compared them with a clinical‐grade DMSO/dextran solution for freezing hematopoietic stem cells. The best post‐thawing cell viability and recovery of viable cells expressing the hematopoietic surface markers CD45^+^ and CD34^+^, as well as superior potency assays, were achieved for one of the assessed DMSO‐free cryopreservation solutions; the use of such solution reach equal or superior performance when compared to the use of DMSO/dextran control solution. However, the other three assessed solutions did not prove to be adequate cryoprotectant solutions for hematopoietic cells. DMSO‐free cryopreservation formulations have yet to showcase universal applicability across a diverse array of cell types, since studies indicate that these approaches yield different benefits for different cell types (Yao and Matosevic 2021).

The influence of ice‐growth direction is frequently underestimated during cryopreservation, which may adversely affect the consistency and quality of the freezing process. Directional freezing enables precise control over ice growth velocity and morphology of ice‐front propagation. Studies exploring this method report mainly positive outcomes (Bahari et al. 2018; M. A. Rodrigues et al. 2013; Si et al. 2010). Specifically, Si et al. (2010) reported that rhesus macaque sperm was effectively cryopreserved using a directional freezing technique, resulting in similar sperm motility compared to those cryopreserved by conventional methods and providing a new and effective method for cell genetic preservation. On the other hand, Bahari et al. (2018) introduced a controlled slow cooling technique that combines initial directional freezing with subsequent gradual cooling down to −80°C for the cryopreservation of adherent cell monolayers on a substrate. Experimental findings revealed 70% of post‐thaw cell viability for Caco‐2 cells frozen under directional ice growth, while cell viability was limited to 15% when a nondirectional freezing approach was used. Rodrigues et al. (2013) have focused on bottom‐up vertical freezing of proteins, which favors the control of ice‐nucleation without significant undercooling, and the formation of an ice structure with a uniform distribution of the concentrated (or glass) phase throughout the ice crystals. By utilizing a bottom‐up freezing geometry, solute concentration variations of less than 10% were achieved throughout the frozen solutions. This method proved reproducible even when the solution volume was scaled‐up by 100‐fold, from 30 mL to 3 L.

Understanding and controlling the direction of ice growth during cryopreservation is critical, as it directly affects mechanical stress experienced by cells and can significantly impact their viability and structural integrity during both freezing and thawing. As water crystallizes, the resulting ice expands and exerts pressure on the surrounding cells, creating a porous matrix and mechanical forces that can disrupt cellular structures (Geraldes et al. 2020). In solutions with low concentrations of cryoprotectants, the ice fraction is high, consisting mostly of pure water. Consequently, the shear stress experienced by cells increases, which can lead to the distortion, rupture, or compression of cellular membranes and organelles, ultimately resulting in cell death. In conventional freezing configurations, an ice crust is typically first formed on air‐liquid interface at the top of the container, and then, when the enclosed liquid freezes, it leads to an increase in internal pressure on the underlayer regions. This elevated pressure forces the liquid phase to move through the porous ice layer towards the air interface, resulting in a higher shear stress experienced by cells that are displaced along with the liquid phase. This phenomenon has previously been documented in 2‐liter and 5‐liter bottles, where the internal pressure reached 10 bar during freezing (Duarte et al. 2020). In a bottom‐up freezing geometry, where the ice grows from the bottom to the top of the container, the liquid is not enclosed, reducing the overall internal pressure inside the container. Nonetheless, to the best of our knowledge, there are few reports addressing the potential benefits of applying this freezing strategy for the cryopreservation of cells (Bahari et al. 2018; M. A. Rodrigues et al. 2013; Si et al. 2010).

The phenomena herein described are inherently difficult to measure experimentally. In the field of cryobiology, several modeling studies have been conducted to investigate slow freezing of animal cells. These efforts have aimed to elucidate the mechanisms underlying osmotic injury, particularly focusing on transmembrane water transport and intracellular ice formation (Hayashi et al. 2021; Hayashi et al. 2022; MAZUR 1963). More recently, computational fluid dynamics (CFD) has been applied to predict cell survival during cryopreservation. Some of these models have incorporated the effects of supercooling, especially relevant in small‐volume systems (Hayashi et al. 2023; Scholz et al. 2024). However, most models still focus primarily on osmotic stress and do not adequately simulate the extracellular microenvironment, including phenomena such as freeze concentration, ice porosity and shear stress. With regard to ice porosity and freeze concentration, CFD has been employed in other fields to simulate and optimize related processes. For instance, Sahasrabudhe et al. (2012) created a numerical model to simulate the freeze concentration of sugarcane juice. The model examined various parameters affecting ice growth and juice concentration. Similarly, Jayakody (2019) developed a CFD model to simulate freeze desalination using liquid nitrogen, which served as a cooling medium to facilitate ice formation and salt separation. While these models represent important advances in fields like food processing and desalination, they do not capture the specific complexities associated with biological systems.

To address some of these challenges, Geraldes et al. (2020) developed a model to simulate convective flow within the porous ice structure and the volume expansion that occurs during freezing. This model enabled accurate prediction of local temperature and solute composition in 0.6 L containers, facilitating the evaluation of potential stresses on biologic stability, including ice porosity, shear stress, and exposure time in a nonideal environment.

In this study, the cryopreservation of hybridoma cells and mononuclear cells (MNCs) derived from human umbilical cord blood (hUCB), using two alternative freezing strategies, bottom‐up freezing method with controlled nucleation and a conventional radial freezing method without controlled nucleation, is assessed in terms of viability (i.e. membrane integrity, for hybridoma and MNCs), as well as immunophenotype and clonogenic potential (for MNCs). CFD simulations were used to estimate the effect of freezing geometry on critical output variables, such as local shear stress and ice porosity during freezing. The CFD tool previously developed in our research group (Geraldes et al. 2020) was applied on this study.

## 2 Materials and Methods

### 2.1 Cell Culture

#### 2.1.1 Hybridoma Cells

Mouse hybridoma cells (cell line AC133.1) were cultured in T‐25 cell culture flasks at a cell seeding density of 1.5×10^5^ cells/mL of CD Hybridoma medium (Gibco, EUA). CD Hybridoma medium was supplemented with 8 mM l‐glutamine (Gibco, EUA) and 1% (v/v) antibiotics (50 U/mL penicillin and 50 μg/mL streptomycin) (Gibco, EUA). Cells were kept at 37°C, 5% CO_2_ and 21% O_2_ and passaged every 3 days until further use. Before each freezing experiment, cells were centrifuged at 1250 rpm for 7 min and resuspended in the cryopreservation medium, prepared at different concentrations of DMSO (Sigma Aldrich, St. Louis, MO).

#### 2.1.2 Human Umbilical Cord Blood (hUCB) Derived Mononuclear Cells (MNCs)

Human umbilical cord blood (hUCB) samples were obtained from healthy donors, after written informed maternal consent according to the Directive 2004/23/EC of the European Parliament and of the Council of 31 March 2004 on setting standards of quality and safety for the donation, procurement, testing, processing, preservation, storage and distribution of human tissues and cells (Portuguese Law 22/2007, June 29), with the approval of the Ethics Committee of Hospital São Francisco Xavier, Centro Hospitalar Lisboa Ocidental, Lisboa, Portugal.

hUCB‐derived MNCs were isolated using Ficoll‐Paque (GE Healthcare, EUA) density gradient centrifugation. Red blood cells were removed with ammonium chloride (Sigma‐Aldrich, St. Louis, MO) lysing solution. The isolated cells were processed and frozen with cryopreservation medium with different DMSO concentrations.

### 2.2 Freezing Methods

**Figure 1:**
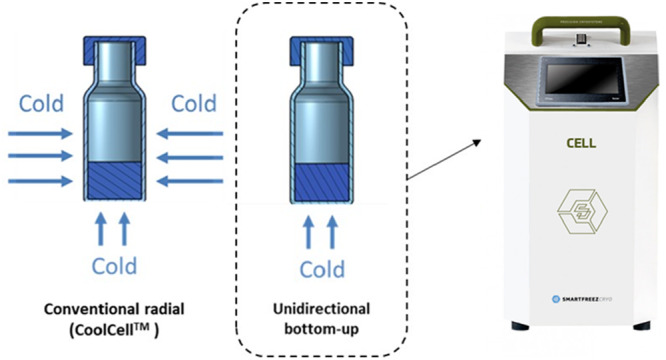
Schematic representation of the heat transfer of the freezing methods using 1 mL glass vials in conventional radial freezing (left) and unidirectional bottom‐up freezing (middle). On the right is shown the CELL developed by SmartFreez, used to obtain a bottom‐up freezing geometry.

The freezing methods were performed using 1 mL glass vials (Thermo Scientific, Waltham, Massachusetts, EUA) with 400 µL of cell suspension. The cryopreservation solutions consisted of Fetal Bovine Serum (FBS) supplemented with 0%, 0.5%, 1%, 2.5%, or 10% DMSO for UCB‐derived MNCs, and CD Hybridoma Medium supplemented with 0%, 1%, 2.5%, 5% or 10% DMSO for hybridoma cells. The two different freezing methods used are graphically described in Figure 1.

For the conventional radial method, the passive cooling device CoolCell^TM^ (Corning, Nova York, EUA) was kept at 4°C before the freezing process. Then, the vials were introduced into the cavities and the device was placed in an ultra‐low freezer (Sanyo, Osaka, Japan) at −80°C for 48 h.

For the bottom‐up freezing process, the CELL controlled‐rate freezer (SmartFreez, Porto Salvo, Portugal) was used. The vials were positioned in a polylactide (PLA) holder and placed within the cooling chamber of the CELL, which features a unique bottom‐up freezing geometry and enables precise ice nucleation control. Initially, the vials were pressed against the cooling surface (filled with a contact fluid), that was previously set at −80°C, for 30 s, forming a thin ice layer at the vial bottom (nucleation step). Afterwards, the temperature was raised to −10°C, followed by a controlled cooling rate of 1°C/min applied to the vial bottoms, gradually reducing the temperature to −80°C. During this controlled rate stage, ice grew from the thin layer of ice formed in the nucleation step towards the top of the vials. These steps are fully automated in the CELL system. Additional figures of the CELL components are available in the supporting files section (Figures S1 and S2). After freezing, the vials were kept in an ultra‐low freezer at −80°C for 48 h.

### 2.3 Thawing Method

Thawing was performed by placing the vials in a water bath at 37°C (Clifton, UK) and gently swirled until there was only a small ice crystal left (2–3 min). After removing the vials from the water bath, fresh culture medium was added (1:10) to dilute DMSO. The cell suspension was centrifuged at 1250 rpm for 7 min, the supernatant was removed and 1 mL of fresh medium was added. Immediately after this procedure, samples were taken to determine cell viability.

### 2.4 Cell Viability and Immunophenotyping

Cell viability was assessed using the Trypan Blue exclusion method, by mixing 0.4% Trypan Blue (Gibco, USA) with the cell suspension to obtain dilution factors of 2 or 20 depending on the initial cell concentration inside the vial. At least three counts were performed per thawed vial using a hemocytometer (MARIENFELD, Germany).

Cell viability was determined as the percentage of cells with intact membranes after freezing and thawing out of the total number of cells present in the cryo‐solution. When freezing higher cell concentrations (10 M cell/mL to 50 M cell/mL), cell loss after freezing was negligible. However, at low cell concentrations (1 M cell/mL), there was a tendency for cell loss, likely due to cell rupture during freezing.

The percentage of apoptotic cells was measured by flow cytometry using Annexin V/propidium iodide (PI) (Thermo Scientific, Waltham, Massachusetts, EUA). Early apoptotic cells are stained with annexin V, but not with PI (annexin V^+^/PI^−^), necrotic cells are stained with annexin V and PI (annexin V^+^/PI^+^) and live cells are neither stained with annexin V nor PI (annexin V^−^/PI^−^).

Immunophenotyping of hUCB‐derived MNCs was performed to assess stem/progenitor cell phenotypes, namely CD34^+^ (expressed by HSPC), as well as CD34^+^CD133^+^ and CD34^+^CD90^+^ (more primitive HSPC populations). Flow cytometric analysis was performed using the following antibodies: CD34‐FITC, CD90‐PE and CD133‐PE with the respective isotype controls (Phycoerythrin (PE) and Fluorescein isothiocyanate (FITC) mouse IgG1), all from BioLegend, California, EUA. Cell samples were prepared at a cell concentration of 1 M cell/mL in 100 µL of phosphate‐buffered saline (PBS) and incubated with the labeled antibodies at room temperature for 20 min protected from light. A minimum of 10,000 events were acquired per sample in a FACSCalibur equipment (Becton Dickinson, EUA). Flow cytometry results were analyzed using FlowJo© software (Becton, Dickinson Company).

### 2.5 Clonogenic Assays

hUCB‐ derived MNCs were washed with Magnetic Activated Cell Sorting (MACS) buffer and CD34^+^ cell selection was performed using a Human CD34 MicroBead Kit UltraPure (Miltenyi Biotec, Germany), following manufacturer's instructions.

HSPCs (i.e. CD34^+^‐enriched cells) were characterized according to their capacity, as stem/progenitor cells, to proliferate and differentiate into different hematopoietic lineages under specific conditions. Cells were resuspended in MethoCult^TM^ methylcellulose‐based medium (Methocult™ GF M3434, STEMCELL Technologies) and seeded on a 12‐well plate in triplicates (total of 10,000 CD34^+^‐enriched cells per UCB sample; cell concentration of 3333 cell/mL per well).

Colony formation was allowed for 14 days at 37°C and 5% CO_2_. Formed colonies [erythroid burst‐forming unit (BFU‐E), colony forming unit granulocyte‐monocyte (CFU‐GM), and colony forming unit granulocyte, erythrocyte, monocyte, megakaryocyte (CFU‐GEMM)] were classified and counted by visual inspection using bright‐field microscopy (Olympus CK40, Japan). Colony numbers were normalized by the number of seeded cells and multiplied by the total nucleated cell (TNC) number.

### 2.6 Computational Fluid Dynamics (CFD) Simulation

The simulations presented in this study were performed using the computational cloud platform SMARTFREEZ*SIM*® with a graphical user interface. The computational model is based on the one described by Geraldes et al. (2020), but instead of an expanding mesh, a Volume of Fluid (VOF) approach was used to include the headspace in the simulation and enable the expansion of the biomixture volume. The previous model already included the transport equations and thermophysical properties of ice and liquid water, as well as other biological formulations. Those equations can be found on the supporting files (Table S1). For the DMSO aqueous solution simulated in this study, the included thermophysical equations are also provided in the supporting files (Table S2).

In more detail, during cell cryopreservation, there is the production of a mushy layer consisting of ice dendrites and interstitial concentrated solutes and cells in equilibrium. Close to the ice front, natural convection occurs due to the concentrated, dense interstitial liquid descending through the low viscous mushy layer. Geraldes et al. (2020) developed a computational model assuming the mushy layer to be a permeable porous medium. The liquid phase composition is determined by the solidification of pure water, which excludes the solutes and increases the concentration of the interstitial aqueous solution. The model also accounts for the increased pressure that pushes the concentrated interstitial solution through the porous ice, a process known as percolation, which is a key mechanism for solute separation from the ice (Aussillous et al. 2006).

To describe the force exerted by the ice matrix on the fluid, a drag force term is included in the momentum equation. This drag force ($F_{\mathrm{mush}}$) is derived from the Kozeny‐Carman equation, which describes the pressure drop of a fluid flowing through a packed bed of solids, as detailed in the previous work of Geraldes et al. (2020) and in Equation 1. In this equation, β is the percolation constant (m^‐2^), µ is the viscosity of the biomixture (Pa.s), α is the volumetric ice fraction and U is the velocity (m/s). The percolation constant is a thermophysical property of the biomixture and is determined by fitting it to the model using the method described by Geraldes et al. (2020). For the simulated DMSO biomixtures, the value of β was 1×10^−10^ m^−2^. The model computes the specific drag force in N/m^3^ of liquid biomixture. Dividing the drag force by the ice porosity will result in the drag force in N/m^3^ of concentrated biomixture ($F_{\mathrm{mush conc})}$ (Equation 2).

(1) $$F_{\mathrm{mush}}=\beta \mu \frac{\alpha^{2}}{{\left(1-\alpha \right)}^{3}}U$$

(2) $$F_{\mathrm{mush conc}=}\frac{F_{\mathrm{mush}}}{1-a}$$

The drag force serves as the basis for calculating the shear stress on the walls of the pore network. This calculation involves scaling the tangential force per unit volume by the pore volume, which depends on the number of pores and their diameter as shown in Equations 3 and 4. The resulting force is then further normalized by the total surface area of the pore walls (Equations 5 and 6), yielding the shear stress (Equation 7). The governing equations are presented below for further clarification.

The shear stress represents the tangential force per unit area exerted by the fluid on the pore walls, as it flows through the interconnected ice pores. Since cells are suspended in the fluid, this stress directly impacts the mechanical and hydrodynamic environment experienced by the cells located within the confined space of the pores, potentially influencing their structural integrity during freezing and thawing processes. To simplify the calculations an average pore diameter of 50 µm was considered. Experimental studies have demonstrated that the pore size in freeze‐dried matrices is highly dependent on the freezing rate, with slower cooling leading to larger and more tortuous pores. Reported values indicate that mannitol‐based formulations can exhibit pore sizes ranging from approximately 20 µm to over 300 µm, while sucrose‐based formulations may reach sizes exceeding 400 µm under specific conditions (Arsiccio et al. 2019; Lammens et al. 2021).

(3) $$V_{\mathrm{pore}}=\frac{\pi }{4}D^{2}L$$

(4) $$V_{\mathrm{total pore}}=n_{\mathrm{pores}}V_{\mathrm{pore}}$$

(5) $$A_{\mathrm{pore}}=\pi \mathrm{DL}$$

(6) $$A_{\mathrm{total pore}}=n_{\mathrm{pores}}A_{\mathrm{pore}}$$

(7) $$Sh\mathrm{ear stress}=\frac{\mathrm{Fmus}h\mathrm{conc}\times V_{\mathrm{total pore}}}{A_{\mathrm{total pore}}}$$

The simulations were performed considering 1 mL glass vials filled with 400 uL of solutions with different DMSO concentrations.

The bottom‐up geometry with controlled nucleation was simulated considering a nucleation time of 30 s. The simulated temperature profile, implemented on the bottom of the vial, was the same as implemented in the CELL equipment. The temperature profile consisted of a plateau at −80°C for 30 s (nucleation step), followed by an increase in temperature to −10°C, and finally a cooling rate of 1°C/min until the temperature reached −80°C. The heat transfer coefficient at the bottom of the vial was assumed to be 150 W.m^−2^.K^−1^, while the lateral walls were assigned a heat transfer coefficient of 5 W.m^−^².K^−1^. These values are typical in process engineering for heat transfer between a liquid and a solid, or between air and a solid under natural convection conditions (Lienhard 2020).

The conventional geometry is designed to achieve a cooling rate of approximately 1°C/min inside the vials, however the heat transfer coefficient on the walls of the vials is unknown. Therefore, before performing the simulations, an experiment was conducted in which all cavities of the CoolCell^TM^ were filled with glass vials, and three thermocouples were strategically positioned to measure temperature at three locations: outside the vial near the lateral wall, outside the vial near the lid, and inside the vial at the center of the solution. Temperature data was recorded over time, and the heat transfer coefficients were adjusted in the CFD model until the simulated temperatures at the liquid center matched the experimental measurements. The detailed results are provided in the supporting materials (Figure S3). Through CFD simulations, the heat transfer coefficient was determined as 3 W.m^‐2^.K^‐1^ on the vial walls. This value was used for all the simulations performed with this freezing geometry.

The ice nucleation temperature, defined as the temperature at which ice formation begins in the simulation, was set to −5°C for all cases.

### 2.7 Statistics

All data are presented as mean ± standard deviation. Statistical analyses were performed using Microsoft Excel and GraphPad Prism (version 10.6.1). Comparisons between two groups were evaluated using Student's *t*‐test, while experiments involving multiple groups were analyzed by one‐way ANOVA followed by Tukey's multiple comparisons test. Differences were considered statistically significant at *p* < 0.05 (*), *p* < 0.01 (**), *p* < 0.001 (***), and *p* < 0.0001 (****).

## 3 Results and Discussion

Based on the findings reported by our group on Rodrigues et al. (2013) regarding cryopreservation of proteins, it was hypothesized that cell viability would vary depending on the freezing geometry employed. Specifically, bottom‐up freezing was compared with conventional radial freezing. To test this hypothesis, a proof‐of‐concept study was conducted using hybridoma cells as a model system, demonstrating notable differences in cell viability between the two geometries. Following this, CFD simulations were employed to investigate the mechanisms underlying these variations. Finally, the study was extended to include hUCB‐derived MNC immunophenotyping using flow cytometry to assess the presence of stem/progenitor cells and gain deeper insights into the observed outcomes. In addition, the clonogenic potential of HSPCs (CD34^+^‐enriched cells) within MNCs was evaluated after cryopreservation.

### 3.1 Effect of Freezing Geometry on Hybridoma Cells Viability: Proof of Concept Experimental Studies

**Figure 2:**
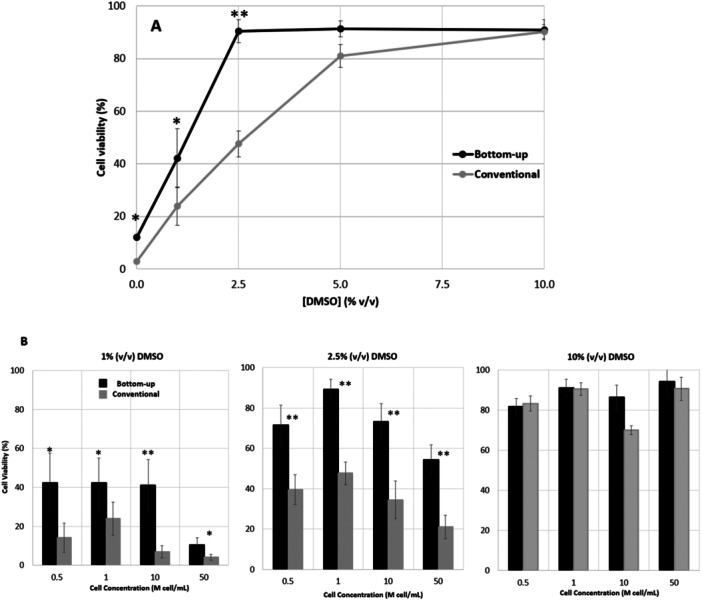
Viability of hybridoma cells after freezing and thawing using the conventional and bottom‐up freezing methods. (A) Cell viability profile using cryo‐solutions with DMSO concentrations between 0% and 10% (v/v), at a starting cell concentration of 1 M cell/mL. (B) Cell viability at cell concentration of 0.5, 1, 10 and 50 M cell/mL using cryo‐solutions with 1, 2.5% and 10% (v/v) DMSO. *p < 0.05, ** *p* < 0.01 values for statistically significant differences. *n* = 2 for 0% DMSO, *n* = 4 for 1% DMSO, *n* = 4 for 2.5% DMSO, *n* = 2 for 5% DMSO, and *n* = 4 for 10% DMSO.

Hybridoma cells, suspended in solutions with varying concentrations of DMSO, ranging from 0% to 10% (v/v), were frozen using the two different freezing geometries to study the impact of ice growth direction and DMSO concentration on cell viability. Cells were initially suspended at a concentration of 1 million cells per mL and frozen with DMSO concentrations of 0%, 1%, 2.5%, 5%, and 10% (v/v) (Figure 2A).

Based on these results, DMSO concentrations of 1%, 2.5%, and 10% (v/v) were selected for further investigation to assess the effect of initial cell concentration, which was varied between 0.5 and 50 million cells per mL (Figure 2B). The 5% (v/v) DMSO condition was excluded from these experiments, as prior observations indicated no significant difference compared to 10% (v/v) DMSO. In general, the results show higher cell viability for the bottom‐up freezing geometry, even though there is a general trend of decreasing viability with lower DMSO concentration (Figure 2A). For both freezing geometries, reducing DMSO concentration below 10% (v/v) does not significantly affect the membrane integrity of hybridoma cells until a critical threshold of DMSO concentration is reached, 2.5% (v/v) DMSO for bottom‐up freezing and 5% (v/v) DMSO for conventional freezing. Below this threshold a noticeable decline in membrane integrity is observed (Figure 2A). The impact of DMSO concentration has been studied for different cell lines and it is visible the existence of a critical DMSO concentration in previously published work. For instance, Murray et al. (2020) observed a 50% reduction in cell survival of mesenchymal stromal cells when utilizing 2.5 (v/v)% DMSO compared to 10% (v/v) DMSO.

The freezing geometry showed a relevant influence on the critical DMSO concentration, below which cell viability dramatically drops. For example, using bottom‐up freezing method for hybridoma cells at a cell concentration of 1 M cell/mL allowed a reduction of DMSO concentration from 10% to 2.5% (v/v), while maintaining high cell viability (90%). Below this threshold, cell viability decreases substantially, suggesting that 2.5% (v/v) DMSO is the critical concentration for these cells using bottom‐up freezing method. In contrast, for the same cells, conventional freezing has a critical concentration of 5% (v/v) DMSO, after which detrimental DMSO concentrations are reached, with cell numbers significantly dropping to only 50% of cell viability at 2.5% (v/v) DMSO concentration (Figure 2A).

Moreover, the effect of the two freezing geometries was investigated for freezing cell concentrations from 0.5 to 50 M cell/mL using three selected DMSO concentrations (1%, 2.5% and 10% (v/v)). Increasing cell concentration is expected to decrease cell viability for systems with lower ice porosity (low concentrations of DMSO). Lower ice porosity reduces the available space for cells to position themselves between ice crystals. As a result, increasing the cell concentration under these conditions can impose additional spatial constraints on the cells. This effect was observed for hybridoma cells, as shown in Figure 2B. Increasing cell concentration by 10 and 50 times was critical for hybridoma cells when DMSO concentration was below 10% (Figure 2B), suggesting that DMSO concentration is related to a cell density limit. The bottom‐up freezing method always provides higher cell viabilities than the conventional method. Still at 1% (v/v) DMSO, those values were limited to 40%; and while, at this low DMSO concentration, such cryoprotectant effect was maintained up to 10 M cell/mL, was not observed when cell concentration increased to 50 M cell/mL. For intermediate DMSO concentrations, at a value of 2.5% (v/v), a parabolic behavior was observed for cell viability as a function of cell concentration, with an optimum viability for a cell concentration of 1 M cell/mL. Note that the cryopreservation of cells at high cell loads is desirable for therapeutical uses and facilitate logistics, indeed cryopreservation protocols recommend the use of 1 to 50 M cell/mL depending on the cell type (Meneghel et al. 2020).

Therapeutic doses for hematopoietic stem/progenitor cell‐based therapies are on the range of 1‐20 million cells per kg of patient (Morgan et al. 2017). Therefore, the results obtained, which achieve vials with viable cells at values of 73% and 86%, respectively for 2.5% and 10% (v/v) DMSO (10 M cells/mL, bottom‐up), is an important achievement. Nevertheless, hybridoma cells are biologically different of hUCB‐derived MNCs, as the later comprise HSPC. Thus, further studies were performed on these conditions using the relevant cell model. Importantly, the use of lower DMSO concentrations is relevant for cell‐based therapies and aligns with clinical safety guidelines, since DMSO can be cytotoxic and may cause adverse effects in patients upon infusion (Fry et al. 2015; Mantri et al. 2015).

In sum, both freezing geometries show a similar trend, although the bottom‐up freezing method enables, using lower DMSO concentrations, nearly two times higher cell viability than conventional freezing method. This result suggests that the ice structure may be less aggressive to the cell viability when ice dendrites grow aligned (bottom to top) instead of concurrently. Bottom‐up freezing is known for inducing more reproducible ice nucleation and achieve more uniform ice structures. However, mechanistically, it is not clear how it contributes to attenuate cell damage (Bahari et al. 2018; Gillis et al. 2022; M. A. Rodrigues et al. 2013). To clarify these mechanisms, we performed CFD simulation to compare the ice porosity and shear stress resulting from the two geometries studied.

### 3.2 Computational Fluid Dynamic Studies (CFD): Insights on Mechanisms

**Figure 3:**
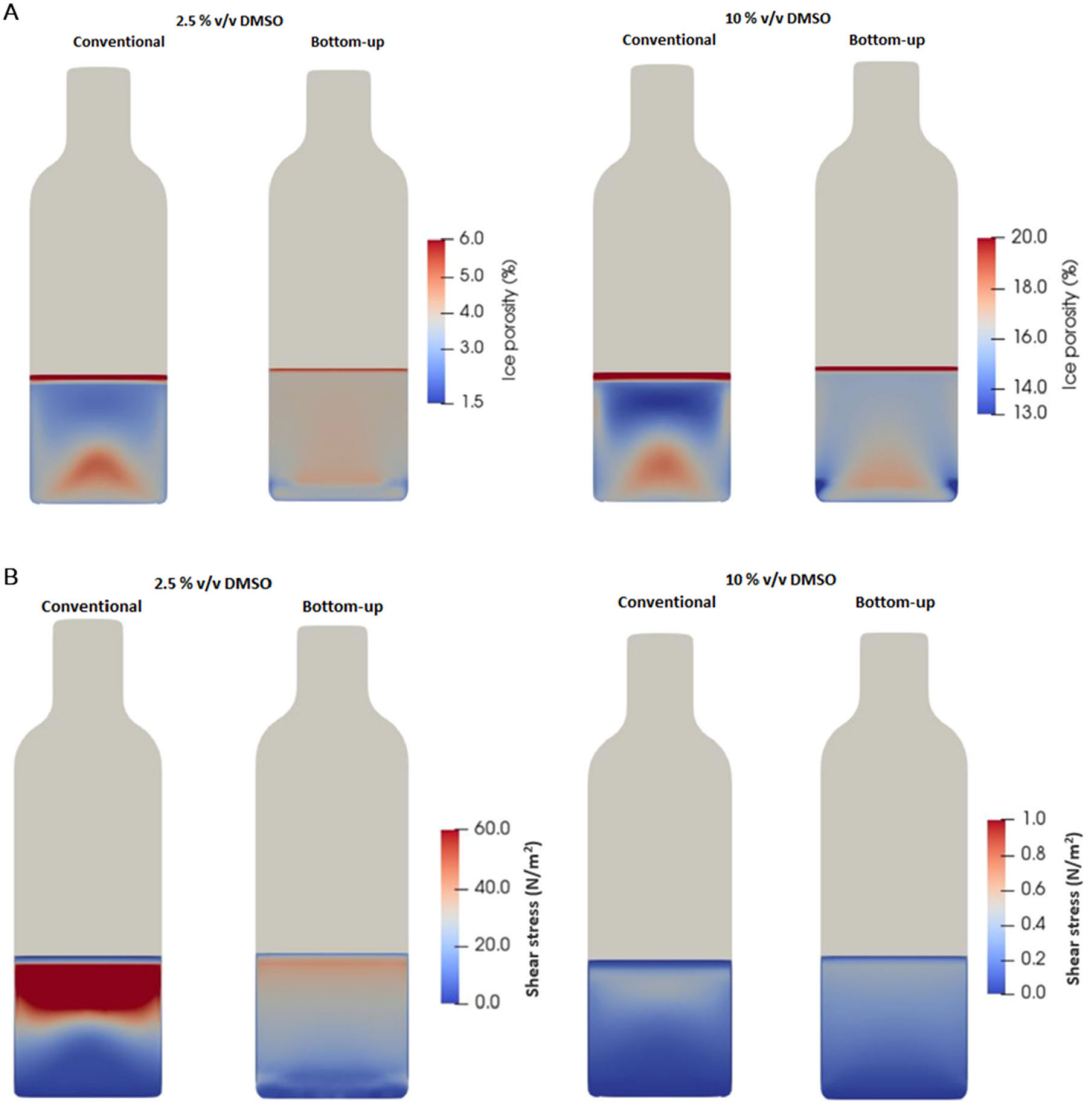
(A) Simulation maps for ice porosity (%) for vials frozen with the bottom‐up freezing with controlled nucleation and conventional method for 2.5% (left) and 10% (v/v) DMSO (right). (B) Shear stress (N/m^2^), for vials frozen with the bottom‐up freezing with controlled nucleation and conventional radial freezing method for 2.5% (left) and 10% (v/v) DMSO (right).

The CFD simulations show that there is not a significant difference concerning the ice porosity between the two geometries when freezing 2.5% and 10% (v/v) DMSO solutions (Figure 3). However, the conventional geometry promotes higher anisotropy, showing ice porosity to be a higher at the center and bottom of the vial and lower on the top of the vial. This heterogeneity is also related to the early freezing of the liquid at the top (at the air interface), enclosing the remaining unfrozen solution. When ice develops at the top, the pressure inside the vial tends to rise, as the enclosed liquid is pushed upwards, generating a resistance to the flow of the liquid that is driven by freezing volume expansion (Duarte et al. 2020). On the other hand, in bottom‐up freezing, the lower porosity region is formed at the bottom, near the ice nucleation layer.

The simulations show that the resistance to the liquid flow caused by the low porosity of the ice at the top can increase by one order of magnitude the shear stresses on the displaced liquid (and cells) frozen in 2.5% (v/v) DMSO concentration using conventional freezing (Figure 3). This effect is not observed for high porous ice formed with 10% (v/v) DMSO. These results align with the observations in Figure 2. For a DMSO concentration of 10% (v/v), both vial geometries maintain cell viability above 80%, with shear stresses throughout the vial remaining below 0.5 N/m². These shear stress levels are consistent with previously reported studies. Grein et al. (2019) demonstrated that the Vero cells used for Measles virus production experience detrimental effects when exposed to shear stresses above 0.25 N/m², but optimal virus titers were achieved at an average shear stress of 0.1 N/m². Zhan et al. (2020) also reported that shear stresses exceeding 7 N/m² led to a 30% reduction in the growth rate of human embryonic kidney cells when compared to those exposed to 1.6 N/m². Shear stress levels that can cause cellular damage have primarily been studied under experimental conditions distinct from those encountered during freezing. Most studies on shear‐induced cell damage are conducted in bioreactors with agitation and aeration, where the mechanical forces differ significantly from those present in freezing environments. In this study, we specifically assess shear stress levels under freezing conditions, recognizing that the thresholds of cellular tolerance may differ from those observed in conventional shear stress experiments.

When the DMSO concentration is reduced to 2.5%, the conventional radial freezing geometry experiences a sharp drop in cell viability from 80% to 50%. In contrast, the bottom‐up geometry maintains viability at 80%. Regarding shear stress, in the conventional geometry with 2.5% DMSO, the estimated shear stress at the top of the vial exceeds 100 N/m². Meanwhile, in the bottom‐up geometry, the estimated shear stress remains below 30 N/m² throughout the container (Figure 3). The ice porosity and shear stress simulation maps for 1% and 5% (v/v) DMSO can be found in the supporting materials (Figure S4).

### 3.3 Effect of Freezing Geometry on Human Umbilical Cord Blood (hUCB)‐Derived Mononuclear Cells (MNCs) Viability: Experimental Studies

**Figure 4:**
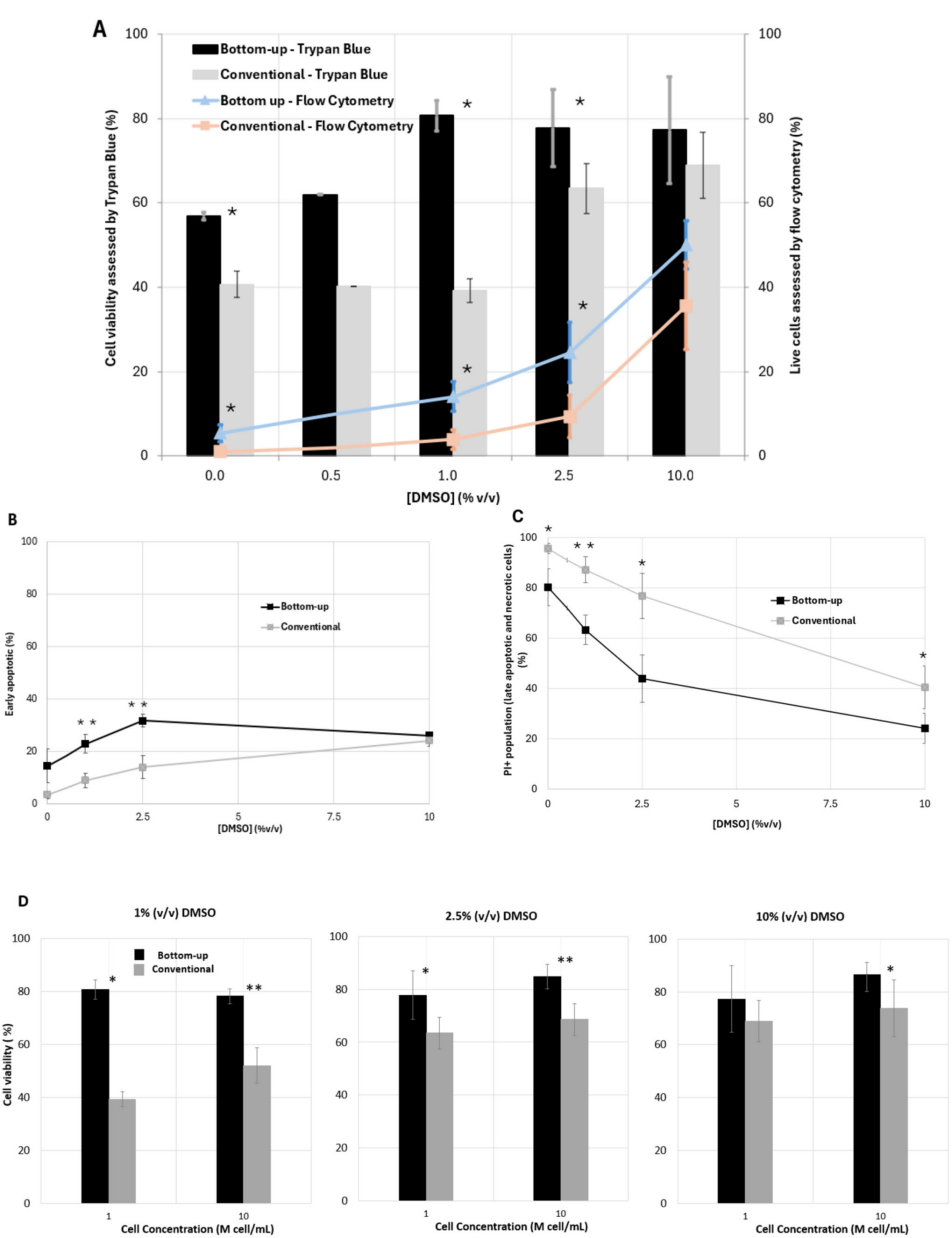
hUCB‐derived MNCs after freezing and thawing using the conventional and bottom‐up freezing methods. (A) Cell viability measured by trypan blue and live cells (Annexin V^‐^/PI^‐^) measured by flow cytometry using cryo‐solutions with DMSO concentrations between 0% and 10% (v/v), at a starting cell concentration of 1 M cell/mL. (B) Early apoptotic cells (Annexin V⁺/PI⁻) measured by flow cytometry using cryo‐solutions with DMSO concentrations between 0% and 10% (v/v). (C) Late apoptotic and necrotic cells (Annexin V⁺/PI⁺ and Annexin V⁻/PI⁺) measured by flow cytometry using cryo‐solutions with DMSO concentrations between 0% and 10% (v/v). (D) Cell viability measured by trypan blue for cell concentrations of 1 and 10 M cell/mL using cryo‐solutions with 1, 2.5% and 10% (v/v) DMSO. *p < 0.05, **p < 0.01 values for statistically significant differences. For cell viability measured with trypan blue, *n* = 4 for 0% DMSO, *n* = 3 for 1% DMSO, *n* = 5 for 2.5% DMSO, and *n* = 5 for 10% DMSO. For FITC‐Annexin V/PI staining, n = 3 for 0% DMSO, *n* = 3 for 1% DMSO, *n* = 3 for 2.5% DMSO, and *n* = 3 for 10% DMSO.

**Figure 5:**
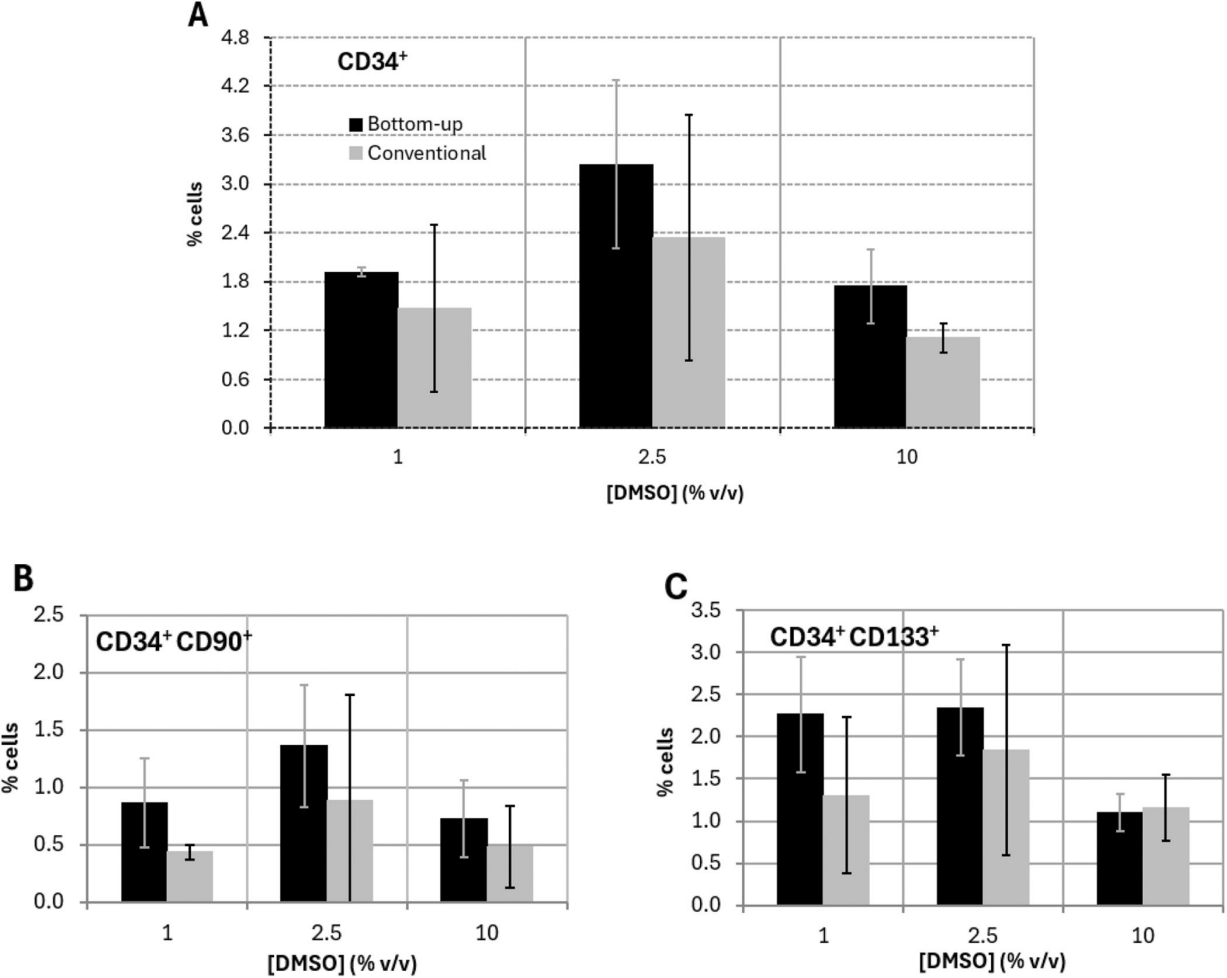
Flow cytometry analysis of hUCB‐derived MNCs with frequencies of (A) CD34^+^ cells on hUCB‐derived MNCs and of (B) CD34^+^CD90^+^ and (C) CD34^+^CD133^+^ cells' subpopulations after freezing and thawing, using the conventional radial freezing and bottom‐up freezing methods. *n* = 3 for 1% DMSO, *n* = 4 for 2.5% DMSO, and *n* = 4 for 10% DMSO.

**Figure 6:**
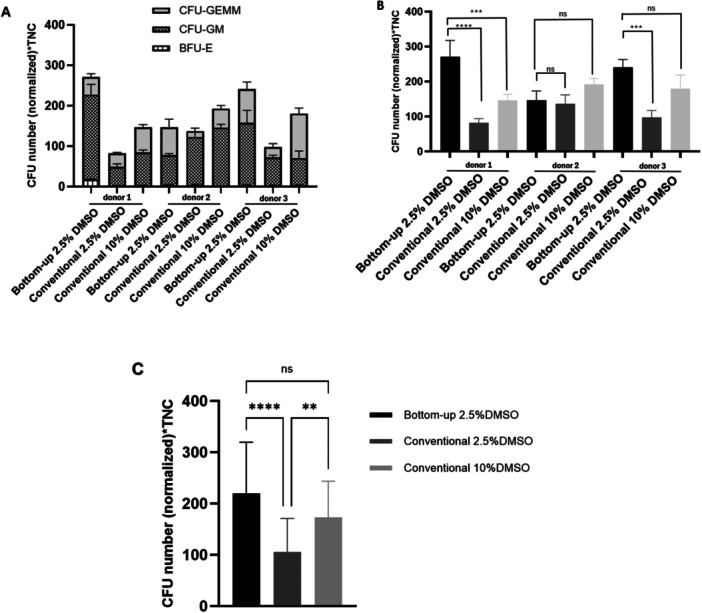
Clonogenic potential of HSPCs (i.e. CD34^+^‐enriched cells) within hUCB‐derived MNCs after cryopreservation using conventional radial or bottom‐up freezing methods. (A) The CFU assay showing numbers of multilineage (CFU‐GEMM), granulocyte/macrophage (CFU‐GM), and erythroid (BFU‐E) colonies generated from HSPCs cryopreserved with bottom‐up freezing (2.5% DMSO) or conventional radial freezing (2.5% or 10% DMSO), across cells from three independent donors. (B) Total CFU numbers per donor (all lineages combined, average of 3 wells each), comparing bottom‐up (2.5% DMSO) with conventional freezing (2.5% or 10% DMSO). (C) Pooled quantification of total CFU numbers (all lineages combined, average of 3 donors) across the three cryopreservation conditions. For all panels, colony numbers were normalized to total nucleated cells. *n* = 3 for both 2.5% and 10% DMSO conditions.

hUCB‐derived MNCs, containing HSPC, are used in transplantation to restore the hematopoietic system, after aggressive treatments of haemato‐oncological diseases. Therefore, their effective cryopreservation is of particular relevance. Considering the data obtained with the use of the hybridoma cells and from the CFD studies, the profile of cell viability after cryopreservation was assessed using selected concentrations of DMSO between 0% and 10% (v/v) at 1 and 10 M cells per mL. In addition, the percentages of live, early apoptotic, late apoptotic, and necrotic hUCB‐derived MNCs, as well as the expression of hematopoietic markers, were characterized by flow cytometry. Furthermore, in vitro clonogenic assays were performed to evaluate the colony‐forming potential of HSPCs (CD34^+^‐enriched cells) within MNCs. Results were obtained after freezing and thawing the cells using the conventional radial and bottom‐up freezing methods, as shown in Figures 4, 5, 6.

Considering cell viability, assessed by Trypan Blue exclusion, the trend observed for hUCB‐derived MNCs was similar to the one observed for hybridoma cells (Figures 2A and 4A), when 1 M cell/mL were cryopreserved. Noteworthy, the use of bottom‐up freezing for hUCB‐derived MNCs allows to achieve the maximum of 80% cell viability with just 1% (v/v) DMSO, a value similar to the one obtained with 10% (v/v) DMSO. The use of a lower concentration of DMSO is particularly relevant for cell‐based clinical applications, contributing to reducing DMSO‐related adverse effects in patients (Bennett et al. 2024; Fry et al. 2015; Mantri et al. 2015). On the other hand, conventional radial freezing requires 2.5% (v/v) DMSO to reach 65% cell viability, almost the same viability level observed at 10% (v/v) DMSO. Similarly, Mantri et al. (2015) investigated the cell viability of UCB‐derived HSPCs post‐freezing with varying DMSO concentrations. Their findings revealed a decline in membrane integrity from 60% to 40% when the DMSO concentration decreased from 5% to 2%, which is consistent with the results obtained in this study.

The maximum cell viability obtained was about 10% lower for the hUCB‐derived MNCs compared to hybridoma cells when freezing 1 M cell/mL at 10% (v/v) DMSO (Figures 2A,B and 4A,D). For both cell models, the bottom‐up freezing geometry allows to use lower DMSO concentrations to reach such maximum values on cell viabilities (1% and 2.5% (v/v) for hUCB‐derived MNCs and for hybridoma cells, respectively, Figures 2A and 4A). At the lower value of 1% (v/v) DMSO, the bottom‐up strategy also led to higher cell viability than the conventional method for both cell models (80% and 43% for hUCB‐derived MNCs and for hybridoma cells, respectively).

Interestingly, for hUCB‐derived MNCs the conventional method exhibited a plateau in cell survival below 1% DMSO, until 0% DMSO (Figure 4A). Similar results were published for the cryopreservation of UCB cells in the absence of DMSO, where the viability assessed by membrane integrity was approximately 50% to 60% using a conventional rate freezer (J. P. Rodrigues et al. 2008). These authors were able to reduce the concentration of DMSO from 10% (v/v) to 2.5% (v/v) by adding trehalose and to 5% (v/v) by adding sucrose, maintaining high cell viability (above 80%).

The percentages of live (Annexin V^‐^/PI^‐^), early apoptotic (Annexin V⁺/PI⁻), late apoptotic (Annexin V⁺/PI^+^), and necrotic cells (Annexin V^‐^/PI^+^) were assessed by flow cytometry as shown in Figure 4 (panels A, B and C), providing a more comprehensive evaluation of cells’ health. The results from FITC‐Annexin V/PI staining showed consistently fewer live cells than those measured by Trypan Blue test analysis (Figure 4A), which is expected given that latter assay assesses only membrane integrity, whereas Annexin V/PI can detect early apoptotic changes before complete membrane disruption. When comparing freezing methods, bottom‐up freezing preserved approximately 15% more live cells than conventional radial freezing for both 10% and 2.5% DMSO. Even at just 1% DMSO, bottom‐up freezing still yielded 10% more viable cells.

Regarding early apoptotic cells, bottom‐up freezing tended to result in about 10% more early apoptotic events compared to conventional freezing (Figure 4B). In contrast, conventional freezing shows a much higher proportion of cells progressing directly to late apoptosis or necrosis, as shown by the PI^+^ population (Figure 4C), bypassing the early apoptotic stage. This may suggest a more abrupt and damaging process in conventional freezing geometry. In the 2.5% DMSO condition, the bottom‐up method results in approximately 45% of late apoptotic and necrotic cells, while conventional freezing leads to around 80% of cells in a late apoptotic or necrotic state. These findings highlight the benefits of reducing DMSO concentration while also emphasizing the importance of optimizing the freezing method. Representative flow cytometry plots corresponding to this analysis have been included in Figure S5 in the supporting files.

Considering clinical applications, where a typical patient requires 150–200 million cells (Morgan et al. 2017; Pasvolsky et al. 2024), the use of 1 M cells/mL may pose logistical challenges. Therefore, freezing 10 million hUCB‐derived MNCs per mL, using 1, 2.5% and 10% (v/v) DMSO, was also investigated (Figure 4D). Again, the trend of the results show that the bottom‐up strategy seems to be superior and could support the cryopreservation of at least up to 10 million hUCB‐derived MNCs per mL without significant loss of cell viability.

Importantly, cryopreservation can differentially affect distinct hematopoietic subpopulations, which can have an effect on both short‐term hematopoietic recovery and long‐term engraftment capacity (Eapen et al. 2020; Maurer et al. 2021). A preliminary flow cytometry analysis was performed to study the effect of cryopreservation conditions on selected cell surface markers (Figure 5). The analysis focused on the relative frequencies of viable HSPCs contained within hUCB‐derived MNCs population, particularly of those expressing CD34, CD133, and CD90 surface markers, across the assays using different DMSO concentrations and freezing geometries. The results indicate that bottom‐up freezing consistently yields higher relative frequencies of CD34^+^CD90^+^ (Figure 5B) and CD34^+^CD133^+^ cells (Figure 5C), commonly associated with early hematopoietic progenitor populations, than conventional radial freezing, across all tested DMSO concentrations. However, these differences were not statistically significant. Representative flow cytometry plots are provided in Supplementary Figure S6.

Assessing colony‐forming unit capacity after cryopreservation is essential, as it captures the proliferative and differentiative potential of HSPCs beyond simple viability measurements. Accordingly, hUCB‐derived HSPCs (i.e. CD34^+^‐enriched cells) were characterized in terms of clonogenic potential following cryopreservation with the bottom‐up freezing method using 2.5% (v/v) DMSO, and with the conventional radial freezing method using either 2.5% (v/v) or 10% (v/v) DMSO (Figure 6). The 2.5% DMSO condition was selected for detailed comparison because, in addition to maintaining high overall viability (above 60% by Trypan blue staining), it revealed clear differences between bottom‐up and conventional freezing in terms of late apoptosis and necrosis (Figure 4C). Although larger statistical differences were observed at 1% DMSO, the 2.5% DMSO condition combines a good post‐thaw viability for both freezing methods with a pronounced apoptotic distinction: in the bottom‐up freezing method, the markers for late apoptosis and necrosis reached 40%, values that are approximately 40% lower than the ones obtained for the conventional radial freezing method. This observation suggests that although cells viability remains relatively high with the conventional freezing method, cells may already be sufficiently damaged to compromise their proliferative potential.

Considering the intrinsic biological variability of hUCB samples, Figure 6A and 6B show that, for cells sourced from two out of the three donors assessed, the bottom‐up freezing method using with 2.5% DMSO produced more colonies, at values approximately 50% and 20% higher, respectively for donor 1 and 3, than when using the conventional radial method with 10% DMSO. When compared with the conventional radial freezing method at 2.5% DMSO, the difference was even more pronounced, with bottom‐up freezing generating more than twice as many colonies. The analysis of the average total colony numbers (CFU‐GM, CFU‐GEMM, and BFU‐E combined) across the cells sourced from the three donors (Figure 6C) further confirmed that bottom‐up freezing with 2.5% (v/v) DMSO consistently yielded higher colony counts. This difference was statistically significant when compared with the conventional radial freezing method under the same DMSO concentration, but not statistically different when comparing with the conventional radial freezing method with 10% DMSO. These results demonstrate that, although cells may appear viable immediately after freezing, their proliferative capacity and colony‐forming ability can be impaired, potentially compromising therapeutic efficacy once thawed and administered to patients. Given that the clonogenic potential of cryopreserved cells was not negatively impacted by the use of a reduced DMSO concentration (2.5%) when the bottom‐up method was applied (relatively to cells cryopreserved with 10% DMSO), this approach may represent a meaningful advantage toward the development of safer and more effective cell‐based therapies. Representative images of colonies after 14 days of culture are shown in Supplementary Figure S7.

### 3.4 Hybridoma and hUCB‐Derived MNCs Viability: Comparison of Experimental and Model Studies

**Figure 7:**
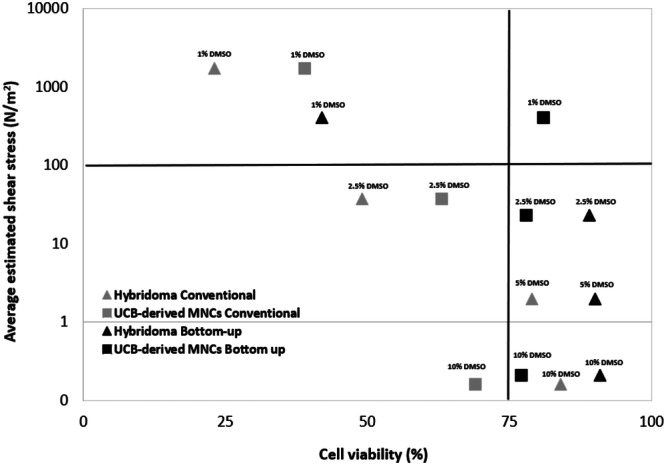
Cell viability, also shown in Figures 2A and 4A, against simulated average shear stress (N/m^2^) for UCB‐derived MNCs and hybridoma cells after freezing and thawing using the conventional radial and bottom‐up freezing methods. The data points are for all the different cryoprotectant concentrations studied: 1% (v/v), 2.5% (v/v), 5% (v/v) and 10% (v/v) DMSO.

The viability results for hUCB‐derived MNCs were plotted alongside those for hybridoma cells (Figures 2A and 4A) against the average estimated shear stress inside the container for each geometry at each DMSO concentration studied, as shown in Figure 7. The data indicates that, with the bottom‐up freezing geometry, most points are positioned on the right side of the graph, corresponding to a region with higher cell viability.

A drop in viability below 40% occurs only in the conventional radial geometry, specifically when the average shear stress exceeds 1000 N/m², a value corresponding to freezing with 1% (v/v) DMSO. Currently, shear stress is considered independent of cell concentration inside the vial. However, our previous experiments have shown that beyond a certain limit, increasing cell concentration can reduce viability due to spatial constraints. Notably, the regions inside the vial with lower porosity, and consequently less available space, also experience higher shear stress. Introducing an additional factor, such as the space occupied by cells, could provide further insights into the interplay between shear stress and cell viability. Future work will focus on establishing a correlation between these variables, incorporating cell concentration as a key parameter. Moreover, different cell types exhibit distinct sensitivities to shear stress. For instance, at estimated shear stress levels above 1000 N/m², hybridoma cell viability drops below 20%, whereas hUCB‐derived MNCs maintain viability around 40%.

These experiments consistently demonstrate a decay in cell viability with decreasing DMSO concentration, which is more accentuated for the conventional radial freezing geometry. When freezing is complete, the ice matrix should then be divided into a fraction of water crystals and a fraction of concentrated DMSO solution, containing the cells. However, we consider that lowering the initial DMSO concentration in conventional radial freezing will decrease the porosity of the ice matrix, exposing cells to mechanical restrictions and higher shear stresses as they become entrapped in ice with lower porosity (Lin et al. 2022), while bottom‐up freezing will mitigate such effects. To further validate and refine these findings, future studies should include direct measurements of ice pore structure, as pore morphology can vary within the container and significantly affect both cell viability and shear stress estimations. Although cell thawing was kept consistent throughout the experiments and it was not the focus of this study, it is important to recognize that ice recrystallization during thawing may also contribute to cell damage. Future studies will thus explore the role of thawing dynamics and how ice crystal morphology, shaped by different freezing geometries, affects cell viability during the thawing process.

## 4 Conclusions

This study highlights the critical role of controlling ice growth direction during cryopreservation. The bottom‐up freezing method consistently yielded higher cells’ viability for both hybridoma cells and UCB‐derived MNCs across a range of DMSO concentrations, outperforming the conventional radial freezing method. Importantly, the bottom‐up freezing method maintained high membrane integrity even at reduced DMSO concentrations (down to 2.5% (v/v) on hybridoma cells and 1% (v/v) on UCB‐derived MNCs).

While the frequencies of key HSPC subpopulations ‐ CD34⁺, CD34⁺CD90⁺, and CD34⁺CD133⁺‐remained comparable across freezing methods, bottom‐up freezing consistently resulted in higher total cell viability and reduced levels of markers for late apoptosis and necrosis. These findings highlight the potential of bottom‐up freezing to enable cryopreservation with lower DMSO concentrations, thereby reducing cytotoxic effects while preserving the functionality of cryopreserved cells. Clonogenic assays have demonstrated that cells cryopreserved using bottom‐up freezing with only 2.5% (v/v) DMSO retain their clonogenic potential comparable to those frozen by the conventional radial freezing method with the standard 10% (v/v) DMSO. Moreover, at the same DMSO concentration of 2.5% (v/v), the bottom‐up freezing method outperforms the conventional radial freezing approach.

The role of ice growth direction on ice porosity and resulting shear stress inside the container were further illustrated through CFD simulations. At high DMSO concentrations (10% (v/v) DMSO), both freezing geometries exhibited similar shear stress profiles. However, at lower DMSO concentrations, the bottom‐up freezing method significantly reduced shear stress (by at least one order of magnitude), demonstrating its importance in mitigating mechanical stress during freezing.

In summary, this study provides valuable insights for optimizing cell cryopreservation protocols, as demonstrated with both hybridoma cells and UCB‐derived MNCs. The bottom‐up freezing method, especially when combined with controlled nucleation, emerges as a promising approach to enhance post‐thawing cell viability and maintain surface marker expression, even under reduced DMSO concentrations (2.5% (v/v)). These findings have important implications for the development of safer and more effective cell‐based therapies.

## Author Contributions

**Rafaela Ouro Neves:** conceptualization, investigation and writing. **Pedro Sena Rego:** conceptualization and investigation. **Marta H. G. Costa:** conceptualization and investigation. **Andreia Duarte:** review and editing. **Isabel Bogalho:** conceptualization and investigation. **Claúdia Lobato da Silva:** supervision, review and editing. **Frederico Castelo Ferreira:** conceptualization, supervision, review and editing. **Vitor Geraldes:** conceptualization, supervision, review and editing. **Miguel A. Rodrigues:** conceptualization, supervision, review and editing.

## Conflicts of Interest

The authors declare no conflicts of interest.

## Supporting information


**Figure S1:** The CELL controlled‐rate freezer is shown on the left. **Figure S2:** 3D model of the holder utilized for freezing vials with the CELL controlled‐rate freezer. **Figure S3:** Experimental temperature profiles (dashed lines) for the freezing of glass vials using the CoolCell™ are presented. **Figure S4:** Top panel: Simulation maps for ice porosity (%) for vials frozen using the bottom‐up freezing method, with controlled nucleation, or the conventional method for 1% (left top panel) and 5% (v/v) DMSO (right top panel). **Figure S5:** Flow cytometry plots from the FITC‐Annexin V/PI staining for the conventional freezing (left panels) and bottom‐up freezing (right panels) methods for DMSO concentrations of 0%, 1%, 2.5% and 10% (top to bottom panels). **Figure S6:** Flow cytometry plots showing the expression of specific surface markers in cells cryopreserved using bottom‐up freezing and conventional freezing methods at three different DMSO concentrations. **Figure S7:** Representative images of colony‐forming unit (CFU) assays of hematopoietic stem and progenitor cells (HSPCs) after cryopreservation with 2.5% (v/v) DMSO using conventional and bottom‐up freezing methods. **Table S1:** Equations of Change and Other Equations Solved by CFD. **Table S2:** Physical Properties of Ice, Liquid Water, and Aqueous Solutions of DMSO. **Table S3:** Nomenclature: Equation symbols
