# Supplementary material for: Bottom‐Up Ice Growth Geometry Attenuates Shear Stress and Improves the Cryopreservation of Hematopoietic Stem/Progenitor Cells Under Low DMSO Concentrations
Source: Biotechnol Bioeng. 2025 Nov 28;123(3):582–97. doi: 10.1002/bit.70116 (PMC12883897; doi:10.1002/bit.70116)
Supplement: Supplementary file 1 — Figure S1: The CELL controlled‐rate freezer is shown on the left. Figure S2: 3D model of the holder utilized for freezing vials with the CELL controlled‐rate freezer. Figure S3: Experimental temperature profiles (dashed lines) for the freezing of glass vials using the CoolCell™ are presented. Figure S4: Top panel: Simulation maps for ice porosity (%) for vials frozen using the bottom‐up freezing method, with controlled nucleation, or the conventional method for 1% (left top panel) and 5% (v/v) DMSO (right top panel). Figure S5: Flow cytometry plots from the FITC‐Annexin V/PI staining for the conventional freezing (left panels) and bottom‐up freezing (right panels) methods for DMSO concentrations of 0%, 1%, 2.5% and 10% (top to bottom panels). Figure S6: Flow cytometry plots showing the expression of specific surface markers in cells cryopreserved using bottom‐up freezing and conventional freezing methods at three different DMSO concentrations. Figure S7: Representative images of colony‐forming unit (CFU) assays of hematopoietic stem and progenitor cells (HSPCs) after cryopreservation with 2.5% (v/v) DMSO using conventional and bottom‐up freezing methods. Table S1: Equations of Change and Other Equations Solved by CFD. Table S2: Physical Properties of Ice, Liquid Water, and Aqueous Solutions of DMSO. Table S3: Nomenclature: Equation symbols [file BIT-123-582-s001.docx]

**Supplementary materials**


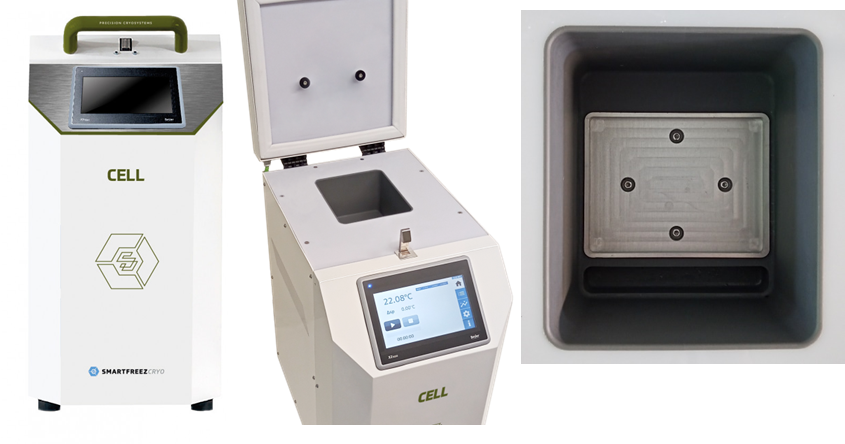


**Figure S1.** The CELL controlled-rate freezer is shown on the left. The center image depicts the CELL with its lid open, while the right image illustrates the cooling chamber and cooling surface of the equipment.


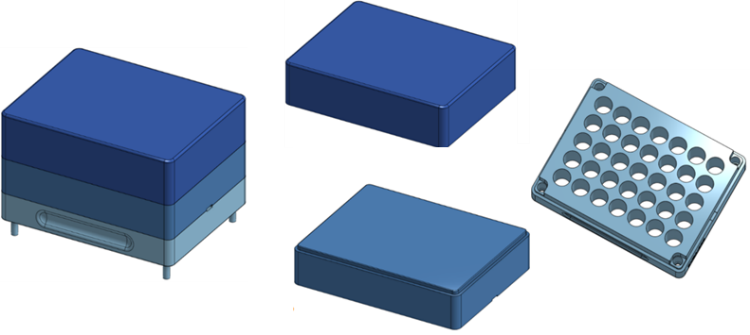


**Figure S2.** 3D model of the holder utilized for freezing vials with the CELL controlled-rate freezer.

**Table S1 :** Equations of Change and Other Equations Solved by CFD

| **Equation name** | **Equation expression** |
| --- | --- |
| **Continuity equation** | $\frac{\delta\rho(1-\alpha)}{\delta t}+\rho_{ice}\frac{\delta a}{\delta t}+\nabla.\left( \rho U_{r} \right)-\rho_{ice}\nabla.\left( \alpha U_{m} \right)=0$ |
| **Momentum equation** | $\frac{\delta\rho U}{\delta t}+\rho\left( U_{r}.\nabla\right)U= \nabla p+ \nabla.\mu\left( \nabla U+\nabla U^{T} \right)+\rho g+F_{mush}$ |
| **Energy equation** | $\frac{\delta\left[ \left( 1-\alpha\right)\rho C_{p}+\rho\alpha C_{p,ice} \right]\left( T-T_{f} \right)}{\delta t}+\nabla.\left( \rho U_{r}C_{p}T \right)-\nabla.\left( \alpha\rho_{ice}U_{m}C_{p,ice}T \right)-\nabla.\left( k\nabla T \right)==T_{f}\nabla.\left( \rho U_{r}C_{p} \right)-T_{f}\nabla\left( \alpha\rho_{ice}U_{m}C_{p,ice} \right)-\nabla.\left( \alpha\rho_{ice}U_{m} \right){\Delta H}_{s}+S_{h}$ |
| **Solute continuity equation** | $\frac{\delta\rho\left( 1-\alpha\right)w_{A}}{\delta t}+\nabla.\left( \rho U_{r}w_{A} \right)-\nabla.\left[ \rho\left( 1-\alpha\right)D_{AB}\nabla w_{A} \right]=0$ |
| **Ice continuity equation** | $\frac{\delta\alpha}{\delta t}=\nabla.\left( U_{m}\alpha\right)+\frac{S_{ice}}{\rho}$ |
| **Liquidus Temperature** | $T_{L}=T_{f}-\frac{K_{f}W_{A}}{M_{A}(1-W_{A})}$ |
| **Ice mass source** | $S_{ice}={\tau\left( 1-\alpha\right)}^{2}\alpha(T-T_{L})(w_{AG}-w_{A})$ |
| **Heat Source** | $S_{h}=S_{ice}{\Delta H}_{s}$ |
| **Momentum Source equation** | $F_{mush}=\beta\mu\frac{\alpha^{2}}{{(1-\alpha)}^{3}}U_{s}$ |

Note: The meaning of the different symbols is shown on the Table S3 on Nomenclature

**Table S2 :** Physical Properties of Ice, Liquid Water, and Aqueous Solutions of DMSO.

| **Physical Properties** | **Equation** |
| --- | --- |
| **Mean heat capacity of ice** | $C_{p,ice}=1116+3.34T, T>190 K$ |
| **Thermal conductivity of ice** | $k_{\mathrm{ice}}=7.47-0.02925T+3.646*{10}^{-5}T^{2}, T>190 K$ |
| **Thermal conductivity of mixture** | $k=\alpha k_{ice}+(1-\alpha)k_{liq}$ |
| **Water density (m^3^/Kg)** | $\rho_{w}=\frac{683.56-64.8058T^{0.5}+1.28265T}{1-{0.127297T}^{0.5}+0.00536447T-0.0000882913}$  $for 233 K<T<300 K$ |
| **Density of aq. sol. (Kg/m^3^)** | $\rho=\frac{1}{w_{A}M_{A}+\left( 1-w_{A} \right)/{\rho_{w}}}$ |
| **Heat transfer in the air interface** | $q_{int}=h\left( T_{int}-T_{air} \right)+\varepsilon\sigma\left( {T_{int}}^{4}-{T_{air}}^{4} \right)$ |
| **Viscosity of aq. sol. of DMSO** | $\mu_{\frac{\mathrm{DMSO}}{\mathrm{Water}}}=$  ${0.00179}^{\left( 36.86-0.234T+0.0003619T^{2} \right)+\left( -4.66+0.0846-0.0002078T \right)w_{A}+\left( 4.82-0.0672T+0.000175T^{2} \right)}$ |
| **Thermal conductivity of solute** | $k_{DMSO}=6.56*{10}^{-4}T+5.68*{10}^{-4}$ |
| **Thermal conductivity of aq. sol. of DMSO** | $k_{\frac{DMSO}{water}}= k_{DMSO}\times wA+k_{water}\left( 1-wA \right)-0.72\vert k_{DMSO}-k_{water}\vert wA\times(1-wA)$ |
| **Apparent specific volume of DMSO (m^3^/Kg)** | $\mathrm{va}_{\mathrm{DMSO}}=\frac{\left( 1\times{10}^{-7}\times T^{0.5} \right)\times\mathrm{Mol}_{a}+(3.85*{10}^{-6}\times T^{0.5})}{\mathrm{MMa}}$  𝑓𝑜𝑟 263 K < 𝑇 < 293 𝐾 |
| **Mean heat capacity of aq. sol. of DMSO** | $C_{p,DMSO/water}=\frac{3.6T-272.01}{MMA*wA}+(1-wA)\times{Cp}_{water}$ |

Note: The meaning of the different symbols is shown on the Table S3 on Nomenclature.


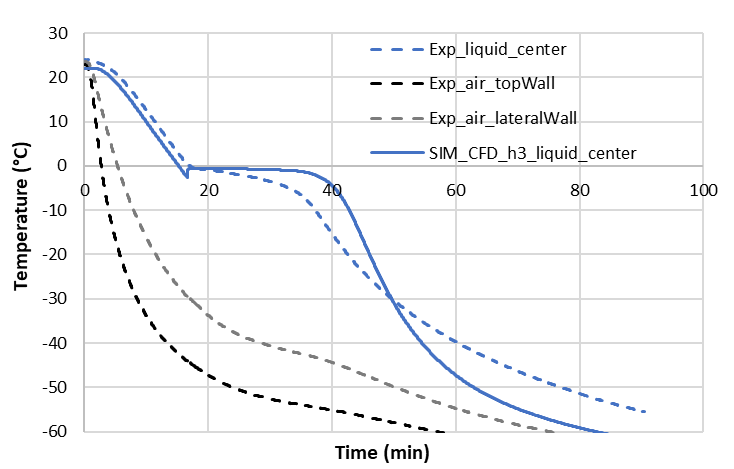


**Figure S3.** Experimental temperature profiles (dashed lines) for the freezing of glass vials using the CoolCell™ are presented. The black dashed curve represents the temperature measured outside the vial near the lid, the grey dashed curve corresponds to the temperature outside the vial near the lateral walls. The blue dashed curve represents the temperature at the liquid center of the solution. The solid blue curve shows the simulated temperature profile at the liquid center of the solution, using a heat transfer coefficient of 3 W.m^-2^.K^-1^ on the defined boundary at the vial walls.


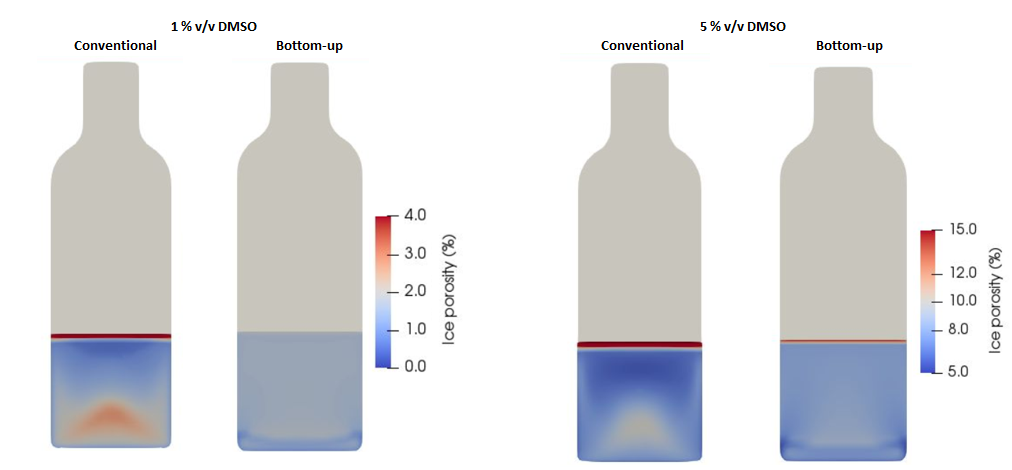


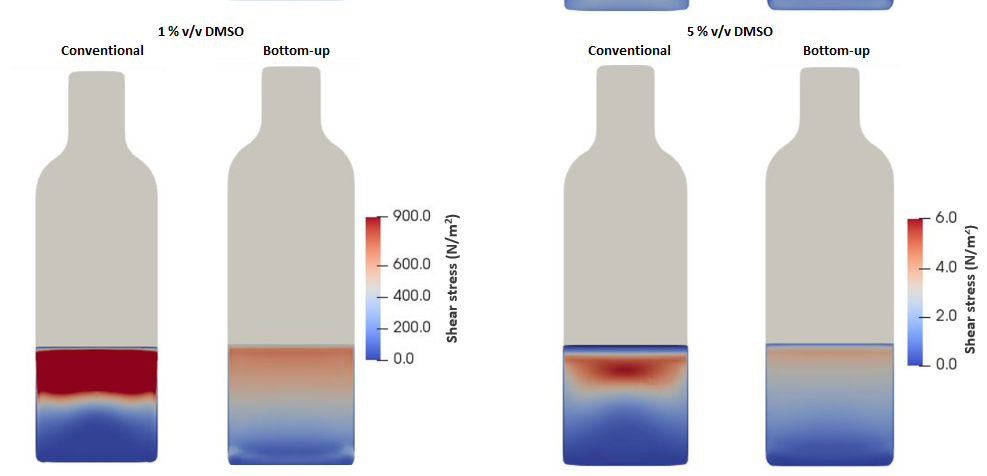


**Figure S4.** **Top panel:** Simulation maps for ice porosity (%) for vials frozen using the bottom-up freezing method, with controlled nucleation, or the conventional method for 1% (left top panel) and 5% (v/v) DMSO (right top panel). **Bottom panel:** Simulation maps of shear stress (N/m^2^) for vials frozen with the bottom-up freezing method with controlled nucleation or the conventional method; for 1% (left bottom panel) and 5% (v/v) DMSO (right bottom panel).

**Figure S5.** Flow cytometry plots from the FITC-Annexin V/PI staining for the conventional freezing (left panels) and bottom-up freezing (right panels) methods for DMSO concentrations of 0%, 1%, 2.5% and 10% (top to bottom panels).


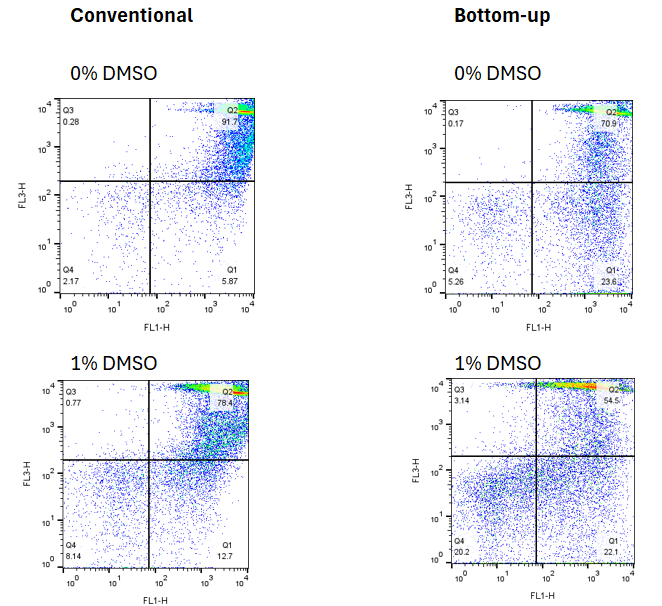

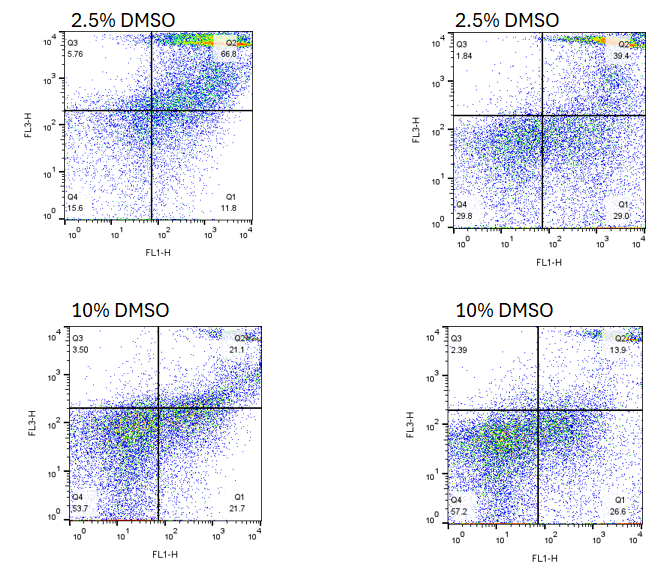


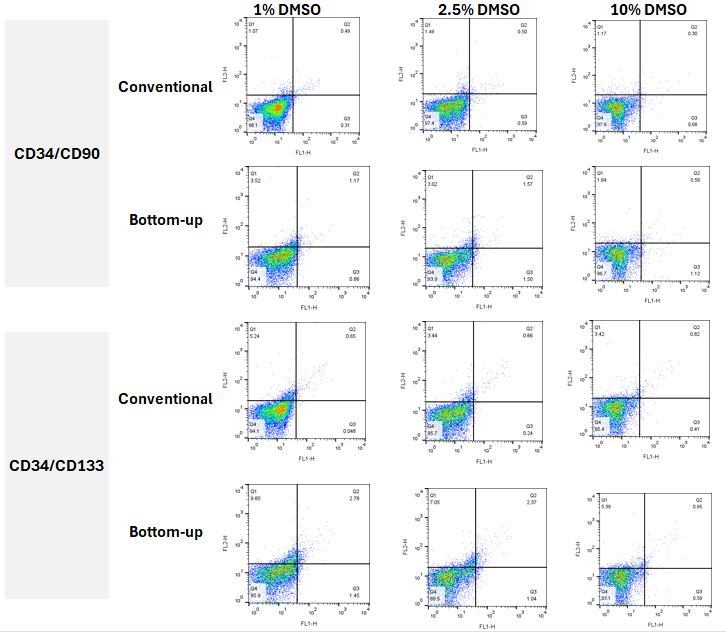


**Figure S6.** Flow cytometry plots showing the expression of specific surface markers in cells cryopreserved using bottom-up freezing and conventional freezing methods at three different DMSO concentrations: 1% (left panels), 2.5% (center panels), and 10% (right panels). **Top panels:** Data for the surface marker CD34/CD90. **Bottom panels:** surface marker CD34/CD133. Each columns show the results obtained at the different DMSO concentration.


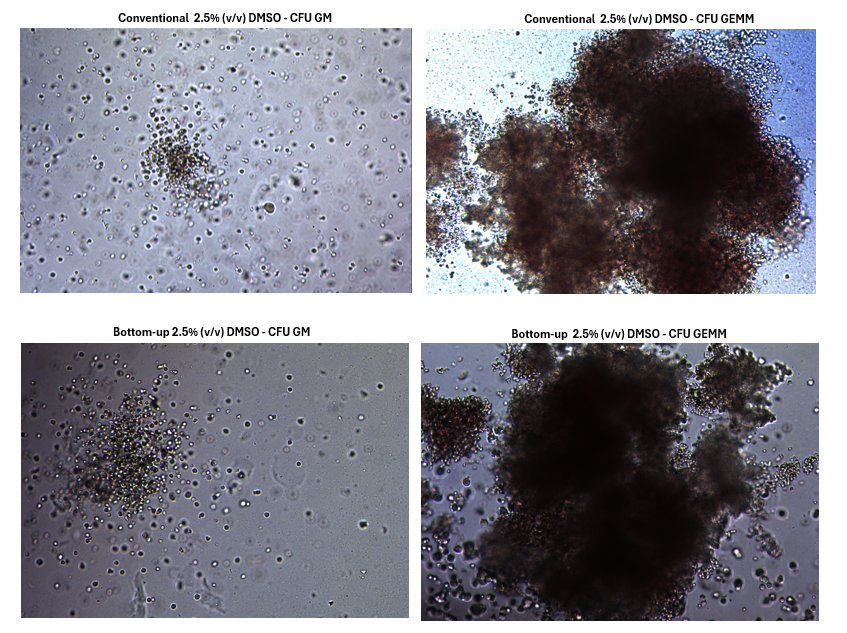


**Figure S7.** Representative images of colony-forming unit (CFU) assays of hematopoietic stem and progenitor cells (HSPCs) after cryopreservation with 2.5% (v/v) DMSO using conventional and bottom-up freezing methods. Colonies were cultured for 14 days in methylcellulose medium. Distinct CFU morphologies were observed, including mixed multilineage colonies (CFU-GEMM) and granulocyte–macrophage colonies (CFU-GM).

**Table S3 :** Nomenclature: Equation symbols

| **Nomenclature:** | |
| --- | --- |
| $w_{A}$ | solute concentration |
| $w_{Ag}$ | glass solute concentration |
| $M_{A}$ | molecular weight (Kg mol^-1^) |
| $K_{f}$ | freezing point depression constant (K mol Kg^-1^) |
| $T_{f}$ | freezing temperature (K) |
| U | velocity of the liquid solution (m s^-1^) |
| $U_{s}$ | superficial velocity (m s^-1^) |
| $U_{r}$ | superficial velocity relative to the mesh motion (m s^-1^) |
| **Greek Symbols:** | |
| *α* | volumetric ice fraction |
| *β* | percolation constant (m^-2^) |
| *ΔH_s_* | enthalpy of solidification of water (333,500 J Kg^-1^) |
| *ε* | emissivity of the liquid-air interface |
| *μ* | viscosity (kg m^-1^ s^-1^) |
| *ρ* | density of the liquid phase (kg m^-3^) |
| *ρ_w_* | density of the water (kg m^-3^) |
| *ρ_ice_* | density of ice (kg m^-3^) |
| *σ* | Stefan-Boltzmann constant (5.67 × 10-8 W m^-2^ K^-4^) |
| *τ* | kinetic constant for ice crystallization (kg m3 s^-1^ K^-1^) |
